# Perceptions in Digital Smile Design: Assessing Laypeople and Dental Professionals’ Preferences Using an Artificial-Intelligence-Based Application

**DOI:** 10.3390/dj12040104

**Published:** 2024-04-11

**Authors:** Smaranda Buduru, Florin Cofar, Anca Mesaroș, Manuela Tăut, Marius Negucioiu, Oana Almășan

**Affiliations:** 1 Prosthetic Dentistry and Dental Materials Department, Iuliu Hațieganu University of Medicine and Pharmacy, 32 Clinicilor Street, 400006 Cluj-Napoca, Romania; dana.buduru@elearn.umfcluj.ro (S.B.); mesaros.anca@elearn.umfcluj.ro (A.M.); oana.almasan@umfcluj.ro (O.A.); 2 Doctoral School, Dental Medicine, Victor Babeş University of Medicine and Pharmacy, 300041 Timișoara, Romania; florin.cofar@dentcof.ro

**Keywords:** Digital Smile Design, artificial intelligence, digital smile planning, dental software, prosthetic dentistry, prosthodontics

## Abstract

Digital Smile Design (DSD) is used in many fields of dentistry. This prospective observational study assessed laypeople’s and dental professionals’ perceptions of a DSD application. SmileCloud, an online DSD platform, was used to create two different designs for three patients; after that, the participants, in a 30-question online illustrated survey, were asked about the most attractive design and other features of the smile. Dentists’ and laypeople’s perceptions about specific DSD features were assessed. The Kolmogorov–Smirnov normality test was used. Descriptive and crosstab analyses compared the respondents’ opinions for each statement. Chi-square tests were used to determine the relationship between the questions and any association with age, gender, and profession. The test results were rated as significant at a *p*-value < 0.05. A total of 520 participants (dental professionals, students, dental technicians, and laypeople) were enrolled. The statistically significant features were self-esteem related to appearance (*p* = 0.05), facial and smile symmetry (*p* = 0.42, *p* < 0.0001), tooth color (*p* = 0.012), and symmetry of gums (*p* < 0.001). For each patient, the design with dominant round upper incisors and perfect symmetry was preferred (*p* < 0.001). Digital pre-visualization benefits diagnosis and enriches treatment planning. The dentist–dental technician–patient team should be involved in the decision-making process of pre-visualization.

## 1. Introduction

Artificial Intelligence (AI) has revolutionized various industries, and dentistry is no exception. In the field of smile design, AI has emerged as a powerful tool that can enhance the accuracy and efficiency of treatment planning, ultimately leading to improved patient outcomes [1].

AI algorithms can analyze vast amounts of data, including patient records, dental images, and facial features, to create personalized smile designs [2]. By considering factors such as facial symmetry, tooth shape, color, and alignment, AI can generate virtual simulations of potential treatment outcomes. This allows dentists to visualize and discuss treatment options with patients, ensuring a more collaborative and informed decision-making process [3].

Moreover, AI can assist in automating the smile design process by suggesting optimal treatment plans based on established dental principles and guidelines [1]. This not only saves time for dentists but also reduces the risk of human error. AI algorithms can also predict the longevity of different treatment options, helping dentists choose the most durable and cost-effective solutions for their patients [2]. One notable example of AI in smile design is the use of machine learning algorithms to analyze facial expressions and emotions. By understanding how different smile designs impact a person’s perceived attractiveness and confidence, AI can help dentists create smiles that not only look aesthetically pleasing but also enhance a patient’s self-esteem [4]. Artificial-intelligence-enhanced smile design software makes it easier to complete a fully digital workflow for Digital Smile Design, a technically difficult treatment process. The use of AI in dentistry is growing rapidly, and a fundamental change triggered by AI is currently taking place [5]. A treatment plan pre-visualization helps both the patient and the dental practitioner to visualize the result of their treatment in a prospective and objective manner. It also enables the patient to approve the procedure before the start of the treatment.

The implementation of a Digital Smile Design workflow is progressively changing today’s dentistry, mainly in the aesthetic and prosthodontics fields. There has been an increase in the use of Digital Smile Design (DSD, smilecloud 2.0, Rubicon (2020)) techniques in numerous branches of dentistry [6]. Communication between dental practitioners and technicians is improved; the workflow is user-friendly and can be operated from any location. The creation of a smile design requires a computer or laptop, intra- and extra-oral photos of the patient, and a general knowledge of dental aesthetics. From the patient’s perspective, the process may seem smoother, more convenient, and less uncomfortable since the protocol includes an intra-oral scanner instead of the conventional impression. Online communication is a significant advantage of this type of workflow. Working online is timesaving and a game changer.

Saving the images on a computer or camera allows the practitioner to go over them without having to recall the patient to the office. Transferring the photos from the camera to the computer increases the odds of keeping them safe. Ethical issues must be considered when employing digitized patient photos for DSD in dentistry. Maintaining patient autonomy and confidence requires adherence to informed consent, privacy, and data protection principles. To practice beneficence and non-maleficence, it is crucial to weigh the possible advantages of DSD against the dangers to patient privacy and data security. Dentists may advance the profession responsibly while also promoting patient well-being by incorporating these ethical considerations into their research and clinical practice in digital dentistry [7]. Moreover, SmileCloud (Version 2.0, Rubicon) is an online cloud-based platform that allows the online storage of all patient data—photos, cone beam computer tomography (CBCT), intra-oral scanning, and functional movement of the mandible—with no limited space, and no risk of data loss.

After all the intra- and extra-oral photographs are collected, they are directly imported to a chosen application where a Digital Smile Design can be simulated. These programs can create highly esthetic and realistic pre-visualizations, including several fundamental esthetic criteria: facial, dento-labial, dental, and gingival esthetics being the most important [8].

After establishing a dental project, the practitioner can make a step forward, allowing the patient to have a tangible pre-visualization of the future prosthetic reconstruction through a digital indirect mock-up. To achieve this aim, the dental technician has to superimpose the Digital Smile Design on the initial intra-oral scan, then print a digital wax-up model and produce a silicone key for the mock-up.

As this is a developing field, several applications are currently available: Digital Smile Design founded by Christian Coachman [9]; Rebel (Visagismile 2.0), a software offered by Galip Gurel [10]; SmileCloud founded by Florin Cofar, a platform that can replace the direct mock-up entirely [11]; and GETapp (Version 1.5.2, 2020) provided by Mauro Fradeani [12]. SmileCloud is an artificial intelligence-based platform that enables the patient and the practitioner to preview various Digital Smile Designs based on realistic photos using natural teeth libraries imported into the application. Based on the unique characteristics of each patient, SmileCloud assists the practitioner in choosing the most appropriate teeth libraries based on the algorithm integrated into the platform. Those aspects make SmileCloud unique and realistic, giving the patient a better understanding of the process. The selected libraries will be superimposed without any distortions by the dental technician to create a digital wax-up [13].

The process of creating a virtual smile design using the SmileCloud application requires at least two pictures of the patient. In the extra-oral picture, the patient sitting upright has to put on a large smile with his mouth wide open. In the intra-oral picture, the lips need to be retracted to visualize the upper arch using the contrastor.

In the context of the continuing evolution of digital resources in dentistry, our study aimed to assess the Digital Smile Design preferences of both laypeople and dental professionals using SmileCloud, a digital pre-visualization tool, prior to initiating a treatment plan; at the same time, we aimed to evaluate the dentists’ and laypeople’s preferences regarding dental, dento-labial, and facial aesthetic parameters. The null hypothesis stated that ideal dental, dento-labial, and facial aesthetic criteria were equally significant in Digital Smile Design and that round teeth shapes and perfect symmetry of the teeth and gingiva are preferred by laypeople and dental professionals.

## 2. Materials and Methods

A prospective, transversal, and observational study on the usefulness of a digital pre-visualization application was carried out among professionals and laypeople. The study was conducted at the Faculty of Dental Medicine, “Iuliu Hațieganu” University of Medicine and Pharmacy, Cluj-Napoca, Romania, only after obtaining the ethics committee’s approval (166/23 June 2022).

The illustrative survey was based on pictures taken in three different clinical cases and the three patients involved in the study signed an informed written consent regarding the use of their photos. The respondents in the survey also agreed and signed an informed consent before being included in the study.

**Figure 1:**
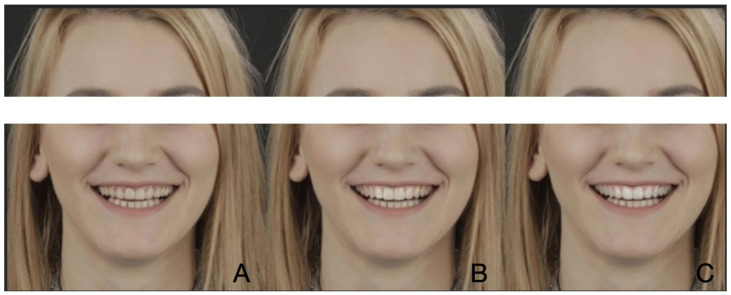
Patient 1: Initial situation (**A**); first pre-visualization (**B**); second pre-visualization (**C**).

**Figure 2:**
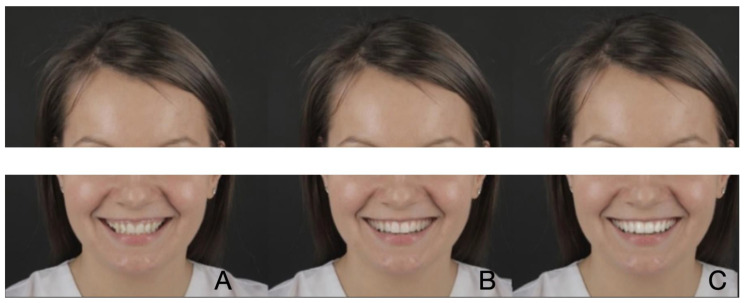
Patient 2: Initial situation (**A**); first pre-visualization (**B**); second pre-visualization (**C**).

**Figure 3:**
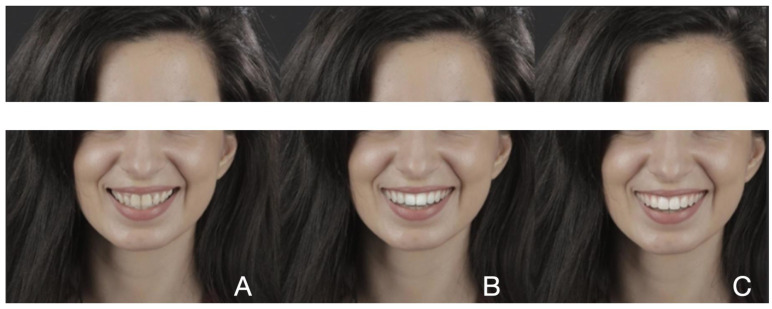
Patient 3: Initial situation (**A**); first pre-visualization (**B**); second pre-visualization (**C**).

For each clinical case, two simulated images of the smile using SmileCloud (Figure 1, Figure 2 and Figure 3) were created. In each case, the first simulated smile featured dominant central upper incisors, open incisal embrasures, ideal tooth proportions with a 75–85% ratio, and perfect symmetry of right and left hemi-arches. Straight posterior teeth axis with a minimal width of buccal corridors and a smile line parallel with the curve of the lower lip were achieved, and a minimal gingival exposure with a pleasant color, shape, and appearance was created. At the same time, guided by the initial appearance of the patient’s smile, all aesthetic parameters were combined to reach a dynamic younger appearance and create a beautiful, but natural smile. The second design of each case also involved ideal tooth proportions, but square shapes, shorter and less dominant central incisors, and a brighter color when compared to the initial situation. The gingival color was also brighter and with medium exposure. Due to the artificial intelligence integrated into SmileCloud, all six designs were created by integrating facial, dento-labial, and tooth aesthetic parameters.

The survey was first randomly pilot-tested among ten subjects to validate the clarity of the questions, the response options, and the time needed for completion. After making the necessary changes, 30 questions were included in the survey (in addition to questions investigating the socio-demographic data).

The questionnaire was distributed between the 4th and the 28th of May 2022 using Google Forms. The inclusion criteria were as follows: students within the Dental Medicine Faculty, dentists, dental technicians, and laypersons. The exclusion criteria resulted in an incomplete data input.

The survey was divided into 3 sections, as follows:

1. The first section included the GDPR agreement so that only the respondents who agreed with the conditions of personal data processing were able to complete the survey.
2. The second section referred to social and demographic factors, such as gender, age, faculty, specialty, and year of study/practice.
3. The third section contained items for assessing the dentists’ and laypeople’s perception about the pre-visualization focusing on facial, dental, and gingival esthetic criteria.

Facial aesthetics were assessed by using a scale and charting the following criteria: facial symmetry; shape and color of the eyes; cheekbones; nose shape and symmetry; the shape of the face; and the appearance of the lips, teeth, chin, and skin. The scale ranged from 1 to 5, with number 1 meaning least important and 5 meaning extremely important.

Smile aesthetics were evaluated by grading the following elements: shape and volume of lips; symmetry of the smile; alignment, visibility, shape, and color of teeth; visibility, appearance, and symmetry of the gums; the width of the maxilla; and visibility of the smile design. The scale ranged from 1 to 5, with 1 meaning least important and 5 meaning extremely important.

The concept of Digital Smile Design and overall aesthetics were evaluated. The significance of appearance for self-esteem, familiarity with DSD, and consequent acceptance of being a DSD patient were among the questions posed to the participants. The scale ranged from 1 to 5, with 1 meaning least important and 5 meaning extremely important.

Finally, we assessed each patient’s most attractive smile. We asked the participants to decide which of the three images (the initial and the two simulations) presented the most attractive smile.

All the answers registered online were further downloaded from the Google Forms website as an Excel spreadsheet. To perform the statistical analysis, the variables were registered, and the answers were exported to an SPSS file (Statistical Package for Social Sciences software 22.0—SPSS, Chicago, IL, USA). The Kolmogorov–Smirnov normality test determined the data distribution. Descriptive and crosstab analyses were used to compare the respondents’ opinions for each statement. To determine the relationship between the questions and any association with age, gender, and profession, Chi-square tests were used. The test results were rated significant at a *p*-value below 0.05.

## 3. Results

The answers from 537 participants were registered initially. After analyzing all the data and applying the inclusion/exclusion criteria, 520 participants’ answers were considered for further statistical analysis. A total of 406 females (78.1%) and 114 males (21.9%) took part in the survey. Most respondents were laypersons, who had no connection with dentistry (*n* = 182; 35.0%);164 subjects were students in the field of dentistry (*n* = 164, 31.5%); 113 respondents were dentists (21.7%); and 61 subjects were dental technicians (11.7%). The age ranged between 18 and 63 years, with most subjects aged between 18 and 30 (*n* = 421, 81%).

### Illustrative Survey

For the third section of the survey, which included the images obtained via SmileCloud, the answers were included in Table 1, Table 2, Table 3 and Table 4. Data were sorted depending on the respondent’s relationship to dentistry, namely via their profession.

Statistically significant results included self-esteem related to appearance; eye shape (3 out of 5 on scale importance) and color (2 out of 5 on scale importance); the appearance of the chin (3 out of 5 on scale importance) and skin (5 out of 5 on scale importance); symmetry of the smile (5 out of 5 on scale importance); teeth color (5 out of 5 on scale importance) and visibility (4 or 5 out of 5 on scale importance); and gums visibility (5 out of 5 on scale importance for dentists, students, and laypeople; 3 out of 5 for dental technicians on scale importance), appearance (5 out of 5 on scale importance for dentists, students, and laypeople; 3 out of 5 for dental technicians on scale importance), and symmetry (4 out of 5 on scale importance for dental technicians and laypeople; 5 out of 5 for dentists and students on scale importance). The appearance, volume, and shape of the lips; the teeth alignment; and maxilla width were not statistically significant.

For the subject featured in Figure 1, the first pre-visualization image was considered the most attractive (*n* = 279; 53.65%), followed by the second pre-visualization (*n* = 228; 43.84%) and the initial situation (*n* = 13; 2.5%). For the subject in Figure 2, the first pre-visualization was considered the most attractive (*n* = 263; 50.57%), followed by the second pre-visualization (*n* = 236; 45.38%) and the initial situation (*n* = 21; 4.03%). For the subject in Figure 3, the first pre-visualization was considered the most attractive (*n* = 260; 50%), followed by the second pre-visualization (*n* = 239; 45.96%) and the initial situation (*n* = 21; 4.03%).

At the beginning of the survey, 67.88% of the participants both not related to dentistry and dental specialists declared that they were familiar with the concept of smile design. However, the number increased to 89.4% (statistically significant) by the end of the survey when asked about the possibility of undergoing a digital wax-up and mock-up for themselves.

## 4. Discussions

The questionnaire answered by 520 participants revealed that several elements are significant when it comes to facial aesthetics. If a pleasing appearance is to be achieved, Digital Smile Design should take the smile as well as facial references into account. SmileCloud platform uses artificial intelligence to guide the designer in respecting facial, dento-labial, and teeth ideal ratio and parameters.

In assessing facial aesthetics, the color and shape of the eyes, even if statistically significant, were not considered to be important in assessing the projects regardless of profession. A previous study has shown that a reference point for determining the appropriate dimension of the buccal corridor might be the distance from the proximal of the pupil and the terminal region of the iris [14]. It has been previously shown that, when employing a lower facial viewpoint, laypeople can accurately distinguish the standard and various degrees of acceptableness [15], which is in accordance with our results, demonstrating how different backgrounds can have fluctuating look-up patterns for a variety of factors. In terms of skin assessment, all the participants considered it extremely important. This may reveal how people interpret beauty and what different individuals are focusing on when asked about analyzing someone’s facial characteristics.

Teeth shade and visibility were of paramount importance for all responders. Gum visibility and appearance seemed to be extremely important for all responders except dental technicians. Numerous studies on gingival appearance have been carried out [16,17]. As previously indicated, the optimal gingival exposure amounts to 2.1 mm, and the admissible variation is approximately 4 mm [8]. When evaluating gingival exposure in smiling, there ought to be 0.4 mm of gum showing [18]. In the present study, we did not measure gingival exposure but focused on gum symmetry and found a consensus—namely, very important for dentists and students and only important for dental technicians and laypeople. The results may derive from the fact that dental technicians are not in direct contact with the pink tissue, whereas the patient’s main concern is linked to the pink and white interface, and most patients believe this to be a potential failure (grey marginal gingivae and dark interdental papillae caused by a metal framework or aluminous ceramic cores in the context of a thin gingival biotype; asymmetrical zeniths for the frontal upper six teeth caused by incorrect preparations or an imprecise finishing line of restorations).

Surprisingly, our results showed that the shape and volume of the lips were not statistically significant—quite a confusing piece of information considering how most dentists use dento-labial references in designing dental composition. On the other hand, hyaluronic acid fillers for lip augmentation are widely spread and used before dental treatment, with increased lip fullness being achieved effectively using injectable hyaluronic acid [19].

Nevertheless, a large number of respondents agreed that teeth alignment was a critical element when assessing a smile. However, smile symmetry was significantly rated by the participants. There was less choice regarding the width of the maxilla, the visibility of the teeth, the symmetry and appearance of the gums, and the visibility of the prosthetics. The respondent’s profession was associated with the visibility of the restoration.

When asked at the beginning of the survey, the number of respondents familiar with the concept of Digital Smile Design was quite high, approximately 67.88%. Yet, the number was even higher (89.4%) when the participants were asked if they considered having a digital mock-up procedure before the onset of the dental treatment.

The tooth visibility and color had a strong statistical significance (*p* < 0.01) for all responders, being the essential parameter in evaluating a pleasant smile. The first design for each case did not suggest a brighter appearance but featured a central incisor dominance that seemed more youthful and dynamic. In this case, one may say that the respondents favored a more genuine smile over a brighter one.

In our study, all the parameters assessing visibility, appearance, and color of the gums seemed as important as the teeth parameters, being statistically significant for all responders. Researchers investigated how dentists, dental students, and laypeople assessed smile designs made by experts or by artificial intelligence (AI) [20]. It has been shown that the use of AI-generated smile designs for symmetrical faces can help practitioners by reducing time and effort in clinical practice [21]. In our study, SmileCloud also received the highest score from dental professionals and students, being a precious time-saving tool for dentists, technicians, and patients alike. It could be stated that the aesthetic gingival parameter, an objective indicator of the health status of the tissue, was appreciated by all responders irrespective of their connection to the dentistry field. It was demonstrated that dental specialists preferred smiles featuring neutral buccal corridor spaces to smiles without such spaces, whereas buccal corridors were not appreciated by laypeople, although they eventually deemed such smiles to be more acceptable [22]. In our study, smile symmetry was statistically significant for all participants, with the highest rate among dentists, followed by students. Responders provided a wide range of responses when asked about interpreting aesthetics and what different people are focused on, although smile symmetry was ascribed to significant importance across all categories. Even if beauty is subjective, symmetry seems to be the main parameter indicating the genuine appearance of the smile. By considering this, the artificial intelligence behind the Digital Smile Design platform enables the designer to work inside strict and well-defined restorative spaces with perfect symmetry and ideal ratios. Numerous efforts have been made to determine lay tolerance limits for smile esthetics; nevertheless, the personal aspect of assessment must be acknowledged, as well as various threshold values for different parameters being evaluated—diastema, tooth size, shape of incisor location, midline disparity, buccal corridors, gingival exposure, and occlusal canting [23]. In our study, dental technicians or patients did not always seem to be always in perfect agreement with dentists or students regarding facial, dental, or gingival aesthetic parameters, which might be an indicator of different perspectives about aesthetics in the dental field. This is why an interdisciplinary Cloud-based Digital Smile Design platform such as SmileCloud should be used to match the dentists’ and laypeople’s perceptions and expectations to improve the outcome of the prosthetic treatment.

One important aspect of the study is the use of an artificial intelligence-based application, SmileCloud, for Digital Smile Design. Following the protocol, constantly expanding the clinicians’ knowledge, and high numbers of analyzed cases and respondents are other significant contributions. Using digital imaging and design has many advantages, one of which is the possibility of visualizing the final treatment result before starting the preparations clinically. In addition, the practitioner can also provide the patients with several designs, giving them the opportunity to choose between different alternatives. It is also a time-saving technique for the dentist, technician, and patient.

Communication between the dentist and the dental technician is also improved. Since everyone has access to the necessary information and can communicate online, a complete treatment can be determined without having to meet in person. Additionally, teeth libraries contain only natural teeth allowing the Digital Smile Design to be a copy of a natural morphology [24]. Communication between the dentist and the patient will strengthen their relationship. Discussing, evaluating the design, and expressing opinions are important in building trust, enabling us to avoid misunderstandings and unexpected results after performing an irreversible procedure.

The procedure of Digital Smile Design would be considered in future treatments by most participants (*n* = 457; 87.88%), whereas 40 subjects were still undecided and 23 would not use it. Participants considered that Digital Smile Design is a good instrument for treatment evaluation, being a good marketing tool and available in all fields of dentistry.

Using pre-visualization software, like the SmileCloud platform, allows patients to actively participate in their treatment plan, increasing motivation and compliance. With pre-visualization and simulation, patients can see what is anticipated. Customizable adjustments enable therapeutic adaptations based on patient preferences. Furthermore, SmileCloud uses realistic and unique genuine tooth libraries to help patients better understand the procedure. Nevertheless, these tools may initially have high setup costs, and their effective operation necessitates extensive training, which raises the learning curve. Interdisciplinary communication is facilitated between the dental technician and the dentist. Data from the literature highlighted how the Digital Smile Design improves dental medicine by providing good communication between doctors and patients, predictable outcomes, and functional and aesthetic restorations that also serve as medico-legal documentation [25]. Protecting intellectual property is essential for safeguarding original photos and preventing illegal duplication.

Digital Smile Design is a futuristic technique that will increase in popularity over time. Despite this, the excessive cost of digital instruments limits many professionals. The time a practitioner must spend learning the application also has an impact on the use of Digital Smile Design. On the other hand, the number of cases and articles related to digital design is constantly growing. However, despite being a modern and newly developed technique, the main purpose is to promote innovative existing programs and to encourage the trend in dentistry [26]. The use of smile documentation enables aesthetic rehabilitation planning from a facial standpoint, increases communication between the practitioner and the patient, and enhances predictable treatment performance [27].

The implications of using a DSD program consist of the ability to show the desired result prior to treatment. The patient participates actively in selecting the teeth’s alignment, color, and shapes in realistic photos. DSD is also used to boost patient motivation to start the treatment. DSD makes interdisciplinary workflow with the dental laboratory and other team members easier, thereby improving dental medicine protocols and providing reliable outcomes, effective dentist–patient communication, and functional and aesthetic restorations that serve as medico-legal documentation [28]. At the same time, using digital patient photos for DSD in dentistry requires careful consideration of ethical issues. Maintaining patient autonomy and confidence requires adherence to the principles of informed consent, privacy, and data protection. In order to practice beneficence and non-maleficence, it is crucial to weigh the possible advantages of DSD against the dangers to patient privacy and data security. Dentists may advance the profession responsibly while promoting patient well-being by incorporating these ethical considerations into their research and clinical practice in digital dentistry.

Based on our results, future studies could assess how patients feel about having their smiles enhanced using a DSD program (SmileCloud). It would be challenging to compare these outcomes with those of other DSD technologies. An in vivo study might additionally emphasize the effects on patients and the results’ long-term durability.

The limitations of this study include the absence of demographic information about the participants, such as group age and place of residence, as well as professional experience (for dental practitioners and dental students). Recognizing the participants’ features could extrapolate the study’s findings and improve the interpretation of the results. Given that the study included only three participants who were comparable in age and gender, it would be wise to do additional research involving participants who are older and have a wider range of dental features. Additional study on a more comprehensive and more varied sample of individuals is needed to confirm and generalize these findings.

## 5. Conclusions

Digital pre-visualization benefits diagnosis and enriches treatment planning in order to provide the patient with the most satisfying smile via the SmileCloud application.

Decision-making in Digital Smile Design should involve the dentist, dental technician, and patient, as they may have varied preferences for the project’s outcome.

The genuine beauty of a smile relies not only on traditional aesthetic characteristics of the teeth but also on the appearance of the gums and symmetry of the lips, chin, skin, and face. Even if dentists’ and laypeople’s perceptions are different regarding aspects the beauty of the smile, the outcome of the treatment should meet everyone’s expectations. For this reason, it is crucial to communicate inside the dentist–dental technician–patient team in the same language, the language of the Digital Smile Design platform.

With a Digital Smile Design tool (SmileCloud), the technician and practitioner must step beyond their comfort zones. They need to achieve a new learning curve, be flexible to cope with each case and meet the patient’s specific needs. This will enable them to provide the most satisfying smile and the most effective treatment results, with all the respondents concluding at the end of the survey that it is a genuinely useful aiding tool in the workflow.

## Figures and Tables

**Table 1:** The answers to the statements of the survey: symmetry and extra-oral features.

	Dental Technician (*n*; %)	Dentist (*n*; %)	Student (*n*; %)	No Connection to Dentistry (*n*; %)	Total (*n*; %)	*p*-Value
Symmetry						0.05
Facial symmetry; scale						0.42
1 (least important)	1; 1.6	2; 1.8	4; 2.4	6; 3.3	13;2.5	
2	2; 3.3	4; 3.5	10; 6.1	13; 7.1	29; 5.6	
3	16; 26.2	15; 13.3	26; 15.9	38; 20.9	95; 18.3	
4	19; 31.1	40; 35.4	66;40.2	62; 34.4	187; 36.0	
5 (extremely important)	23; 37.7	52; 46.0	58; 35.4	63; 34.6	196; 37.7	
Nose symmetry; scale						0.56
1 (least important)	2; 3.3	2; 1.8	4; 2.4	7; 3.8	15; 2.9	
2	5; 8.2	3; 2.7	10; 6.1	13; 7.1	31; 6.0	
3	11; 18.0	14; 12.4	30; 18.3	29; 15.9	84; 16.2	
4	18; 29.5	39; 34.5	49; 29.9	68; 37.4	174; 33.5	
5 (extremely important)	25; 41.0	55; 48.7	71; 43.3	65; 35.7	216; 41.5	
Smile symmetry; scale						<0.0001
1 (least important)	0; 0	3; 2.7	1; 0.6	5; 2.7	9; 1.7	
2	1; 1.6	1; 0.9	4; 2.4	8; 4.4	14; 2.7	
3	10; 16.4	5; 4.4	23; 14.0	42; 23.1	80; 15.4	
4	15; 24.6	24; 21.2	46; 28.0	64; 35.2	149; 28.7	
5 (extremely important)	35; 57.4	80; 70.8	90; 54.9	63; 34.6	268; 51.5	
Cheekbones; scale						0.123
1 (least important)	3; 4.9	3; 2.7	10; 6.1	13; 7.1	29; 5.6	
2	17; 27.9	22; 19.5	22; 13.4	32; 17.6	93; 17.9	
3	25; 41.0	36; 31.9	66; 40.2	64; 35.2	191; 36.7	
4	11; 18.0	43; 38.1	45; 27.4	51; 28.0	150; 28.8	
5 (extremely important)	5; 8.2	9; 8.0	21; 12.8	22; 12.1	57; 11.0	
Nose shape; scale						0.07
1 (least important)	3; 4.9	2; 1.8	2; 1.2	5; 2.7	12; 2.3	
2	9; 14.8	8; 7.1	7; 4.3	9; 4.9	33; 6.3	
3	16; 26.2	17; 15.0	33; 20.1	44; 24.2	110; 21.2	
4	17; 27.9	49; 43.4	64; 39.0	61; 33.5	191; 36.7	
5 (extremely important)	16; 26.2	37; 32.7	58; 35.4	63; 34.6	174; 33.5	
Appearance of lips; scale						0.29
1 (least important)	1; 1.6	1; 0.9	3; 1.8	4; 2.2	9; 1.7	
2	5; 8.2	4; 3.5	9; 5.5	11; 6.0	29; 5.6	
3	19; 31.1	16; 14.2	30; 18.3	38; 20.9	103; 19.8	
4	16; 26.2	47; 41.6	71; 43.3	74; 40.7	208; 40.0	
5 (extremely important)	20;32.8	45; 39.8	51; 31.1	55; 30.2	171; 32.9	
Appearance of chin; scale						0.04
1 (least important)	0; 0	1; 0.9	5; 3.0	10; 5.5	16; 3.1	
2	8; 13.1	7; 6.2	21; 12.8	25; 13.7	61; 11.7	
3	27; 44.3	33; 29.2	59; 36.0	63; 34.6	182; 35.0	
4	19; 31.1	44; 38.9	47; 28.7	57; 31.3	167; 32.1	
5 (extremely important)	7; 11.5	28; 24.8	32; 19.5	27; 14.8	94; 18.1	
Appearance of skin; scale						0.05
1 (least important)	6; 9.8	8; 7.1	5; 3	6; 3.3	25; 4.8	
2	4; 6.6	5; 4.4	11; 6.7	11; 6.0	31; 6.0	
3	13; 21.3	19; 16.8	45; 27.4	32; 17.6	109; 21.0	
4	13; 21.3	48; 4.5	46; 28.0	46; 25.3	153; 29.4	
5 (extremely important)	25; 41.0	33; 29.2	57; 34.8	87; 47.8	202; 38.8	
Shape of the face; scale						0.06
1 (least important)	3; 4.9	5; 4.4	7; 4.3	9; 4.9	24; 4.6	
2	7; 11.5	9; 8.0	10; 6.1	22; 12.1	48; 9.2	
3	20; 32.8	30; 26.5	54; 32.9	54; 29.7	158; 30.4	
4	8; 13.1	43; 38.1	50; 30.5	62; 34.1	163; 31.3	
5 (extremely important)	23; 37.7	26; 23.0	43; 26.2	35; 19.2	127; 24.4	
Shape of the lips; scale						0.41
1 (least important)	2; 3.3	1; 0.9	3; 1.8	4; 2.2	10; 1.9	
2	5; 8.2	2; 1.8	13; 7.9	11; 6.0	31; 6.0	
3	16; 26.2	23; 20.4	34; 20.7	50; 27.5	123; 23.7	
4	20; 32.8	53; 46.9	70; 42.7	64; 35.2	207; 39.8	
5 (extremely important)	18; 29.5	34; 30.1	44; 26.8	53; 29.1	149; 28.7	
Volume of the lips; scale						0.85
1 (least important)	3; 4.9	1;0.9	4;2.4	5; 2.7	13; 2.5	
2	6; 9.8	8; 7.1	13; 7.9	18; 9.9	45; 8.7	
3	19; 31.1	28; 24.8	46; 28.0	55; 30.2	148; 28.5	
4	19; 31.1	48; 42.5	67; 40.9	64; 35.2	198; 38.1	
5 (extremely important)	14; 23.0	28; 24.8	34; 20.7	40; 22.0	116; 22.3	

**Table 2:** The answers to the statements of the survey: Digital Smile Design concept.

	Dental Technician (*n*; %)	Dentist (*n*; %)	Student (*n*; %)	No Connection to Dentistry (*n*; %)	Total (*n*; %)	*p*-Value
Do you consider that appearance may influence self-esteem?						0.05
Maybe	7; 11.5	8; 7.1	9; 5.5	24; 13.2	48; 9.2	
No	1; 1.6	0; 0	1; 0.6	5; 2.7	7; 1.3	
Yes	53; 86.9	105; 92.9	154; 93.9	153; 84.1	465; 89.4	
Decision to undergo Digital Smile Design procedures as a patient after the SmileCloud pre-visualizations						0.002
No	4; 6.6	2; 1.8	11; 6.7	6; 3.3	23;4.4	
Still undecided	5; 8.2	1; 0.9	10; 6.1	24; 13.2	40; 7.7	
Yes	52; 85.2	110; 97.3	143; 87.2	152; 83.5	45; 87.9	
Digital Smile Design visibility; scale						<0.0001
1 (least important)	1; 1.6	4; 3.5	9; 5.5	13; 7.1	27; 5.2	
2	2; 3.3	2; 1.8	15; 9.1	24; 13.2	43; 8.3	
3	8; 13.1	16; 14.2	21; 12.8	52; 28.6	97; 18.7	
4	27; 44.3	27; 23.9	48; 29.3	46; 25.3	148; 28.5	
5 (extremely important)	23; 37.7	64; 56.6	71; 43.3	47; 25.8	205; 39.4	
Digital Smile Design concept familiarity						<0.0001
Maybe	5; 8.2	8; 7.1	9; 5.5	33; 18.1	55; 10.6	
No	2; 3.3	7; 6.2	19; 11.6	84; 46.2	112; 21.5	
Yes	54; 88.5	98; 86.7	136; 82.9	65; 35.7	353; 67.9	
Digital Smile Design procedure before undergoing treatment						0.09
Maybe	2; 3.3	5; 4.4	6; 3.7	17; 9.3	30; 5.8	
No	1; 1.6	3; 2.7	10; 6.1	11; 6.0	25; 4.8	
Yes	58; 95.1	105; 92.9	148; 90.2	154; 84.6	465; 89.4	

**Table 3:** The answers to the statements of the survey: the most attractive smile.

	Dental Technician (*n*; %)	Dentist (*n*; %)	Student (*n*; %)	No Connection to Dentistry (*n*; %)	Total (*n*; %)	*p*-Value
Figure 1						
Middle	41; 67.2	70; 61.9	95; 57.9	73; 40.1	279; 53.7	<0.0001
Left	1; 1.6	0; 0	2; 1.2	10; 5.5	13; 2.5	
Right	19; 31.1	43; 38.1	67; 40.9	99; 54.4	228; 43.8	
Figure 2						0.01
Middle	39; 63.9	67; 59.3	85; 51.8	72; 39.	263; 50.6	
Left	1; 1.6	0; 0	11; 6.7	9; 4.9	21; 4.0	
Right	21; 34.4	46; 40.7	68; 41.5	101; 55.5	236; 45.4	
Figure 3						0.03
Middle	25; 41.0	47; 41.6	83; 50.6	105; 57.7	260; 50.0	
Left	4; 6.6	2; 1.8	6; 3.7	9; 4.9	21; 4.0	
Right	32; 52.5	64; 56.6	75; 45.7	68; 37.4	239; 46.0	

**Table 4:** The answers to the statements of the survey: intra-oral items—teeth appearance, alignment, visibility, color, shape, gums visibility, appearance, symmetry, and maxilla width.

	Dental Technician (*n*; %)	Dentist (*n*; %)	Student (*n*; %)	No Connection to Dentistry (*n*; %)	Total (*n*; %)	*p*-Value
Appearance of teeth; scale						0.23
1 (least important)	1; 1.6	3; 2.7	2; 1.2	1; 05	7; 1.3	
2	1; 1.6	0; 0	2; 1.2	1; 0.5	4; 0.8	
3	3; 4.9	1; 0.9	7; 4.3	9; 4.9	20; 3.8	
4	15; 24.6	19; 16.8	35; 21.3	53; 29.1	122; 23.5	
5 (extremely important)	41; 67.2	90; 79.6	118; 72.0	118; 64.8	367; 70.6	
Teeth alignment; scale						0.12
1 (least important)	0; 0	2; 1.8	2; 1.2	2; 1.1	6; 1.2	
2	3; 4.9	2; 1.8	3; 1.8	3; 1.6	11; 2.1	
3	2; 3.3	3; 2.7	9; 5.5	22; 12.1	36; 6.9	
4	16; 26.2	35; 31.0	53; 32.3	59; 32.4	163; 31.3	
5 (extremely important)	40; 65.6	71; 62.8	97; 59.1	96; 5.7	304; 58.5	
Teeth visibility; scale						<0.0001
1 (least important)	0; 0	1; 0.9	2; 1.2	3; 1.6	6; 1.2	
2	1; 1.6	2; 1.8	9; 5.5	12; 6.6	24; 4.6	
3	12; 19.7	5; 4.4	22; 13.4	41; 22.5	80; 15.4	
4	26; 42.6	34; 30.1	68; 41.5	50; 27.5	178; 34.2	
5 (extremely important)	22; 36.1	71; 62.8	63; 38.4	76; 41.8	232; 44.6	
Teeth color; scale.						0.012
1 (least important)	0; 0	2; 1.8	1; 0.6	4; 2.2	7; 1.3	
2	1; 1.6	2; 1.8	3; 1.8	2; 1.1	8; 1.5	
3	5; 8.2	16; 14.2	12; 7.3	12; 6.6	45; 8.7	
4	16; 26.2	43; 38.1	72; 43.9	48; 26.4	179; 34.4	
5 (extremely important)	39; 63.9	50; 44.2	76; 46.3	116; 63.7	281; 54.0	
Teeth shape; scale						0.79
1 (least important)	1; 1.6	1; 0.9	2; 1.2	3; 1.6	7; 1.3	
2	5; 8.2	3; 2.7	6; 3.7	8; 4.4	22; 4.2	
3	13; 21.3	16; 14.2	26; 15.9	35; 19.2	90; 17.3	
4	20; 32.8	48; 42.5	71; 43.3	65; 35.7	204; 39.2	
5 (extremely important)	22; 36.1	45; 39.8	59; 36.0	71; 39.0	197; 37.9	
Gums visibility; scale						<0.0001
1 (least important)	3; 4.9	2; 1.8	5; 3.0	14; 7.7	24; 4.6	
2	8; 13.1	6; 5.3	9; 5.5	25; 13.7	48; 9.2	
3	17; 27.9	10; 8.8	38; 23.2	42; 23.1	107; 20.6	
4	16; 26.2	35; 31.0	48; 29.3	45; 24.7	144; 27.7	
5 (extremely important)	17; 2.9	60; 53.1	64; 39.0	56; 30.8	197; 37.9	
Gums appearance; scale						<0.0001
1 (least important)	1; 1.6	3; 2.7	3; 1.8	9; 4.9	16; 3.1	
2	4; 6.6	6; 5.3	8; 4.9	21; 11.5	39; 7.5	
3	21; 34.4	16; 14.2	31; 18.9	50; 27.5	118; 22.7	
4	20; 32.8	28; 24.8	46; 28.0	49; 26.9	143; 27.5	
5 (extremely important)	15; 24.6	60; 53.1	76; 46.3	53; 29.1	204; 39.2	
Gums symmetry; scale						<0.0001
1 (least important)	0; 0	2; 1.8	6; 3.7	12; 6.6	20; 3.8	
2	6; 9.8	3; 2.7	15; 9.1	25; 13.7	49; 9.4	
3	15; 24.6	14; 12.4	33; 20.1	52; 28.6	114; 21.9	
4	23; 37.7	36; 31.9	48; 29.3	51; 28.0	158; 30.4	
5 (extremely important)	17; 27.9	58; 51.3	62; 37.8	42; 23.1	179; 34.4	
Maxilla width; scale						0.194
1 (least important)	0; 0	1; 0.9	6; 3.7	7; 3.8	14; 2.7	
2	7; 11.5	7; 6.2	13; 7.9	17; 9.3	44; 8.5	
3	23; 37.7	24; 21.2	48; 29.3	58; 31.9	153; 29.4	
4	20; 32.8	49; 43.4	64; 39.0	68; 37.4	201; 38.7	
5 (extremely important)	11; 18.0	32; 28.3	33; 20.1	32; 17.6	108; 20.8	

## Data Availability

The raw data supporting the conclusions of this article will be made available by the authors on request.
